# Interleukin 27 is increased in carotid atherosclerosis and promotes NLRP3 inflammasome activation

**DOI:** 10.1371/journal.pone.0188387

**Published:** 2017-11-27

**Authors:** Ida Gregersen, Øystein Sandanger, Erik T. Askevold, Ellen Lund Sagen, Kuan Yang, Sverre Holm, Turid M. Pedersen, Mona Skjelland, Kirsten Krohg-Sørensen, Trond Vidar Hansen, Tuva Børresdatter Dahl, Kari Otterdal, Terje Espevik, Pål Aukrust, Arne Yndestad, Bente Halvorsen

**Affiliations:** 1 Research Institute of Internal Medicine, Oslo University Hospital Rikshospitalet,Oslo, Norway; 2 Faculty of Medicine, University of Oslo Oslo, Norway; 3 K.G. Jebsen Inflammatory Research Center, University of Oslo, Oslo, Norway; 4 Center for Heart Failure Research, University of Oslo, Oslo, Norway; 5 Department of Neurology Oslo University Hospital Rikshospitalet, Oslo, Norway; 6 Department of Thoracic and Cardiovascular Surgery, Oslo University Hospital Rikshospitalet, Oslo, Norway; 7 Department of Pharmaceutical Chemistry, School of Pharmacy, University of Oslo, Oslo, Norway; 8 Centre of Molecular Inflammation Research, Department of Cancer Research and Molecular Medicine, Norwegian University of Science and Technology, Trondheim, Norway; 9 Section of Clinical Immunology and Infectious Diseases, Oslo University Hospital Rikshospitalet, Oslo, Norway; Virginia Polytechnic Institute and State University, UNITED STATES

## Abstract

**Aim:**

Interleukin-27 (IL-27) is involved in different inflammatory diseases; however, its role in atherosclerosis is unclear. In this study we investigated the expression of IL-27 and its receptor in patients with carotid atherosclerosis and if IL-27 could modulate the inflammatory effects of the NLRP3 inflammasome *in vitro*.

**Methods:**

Plasma IL-27 was measured by enzyme immunoassay in patients with carotid stenosis (n = 140) and in healthy controls (n = 19). Expression of IL-27 and IL-27R was analyzed by quantitative PCR and immunohistochemistry in plaques from patients and in non-atherosclerotic vessels. THP-1 monocytes, primary monocytes and peripheral blood mononuclear cells (PBMCs) were used to study effects of IL-27 *in vitro*.

**Results:**

Our main findings were: (i) Plasma levels of IL-27 were significantly elevated in patients with carotid atherosclerotic disease compared to healthy controls. (ii) Gene expression of IL-27 and IL-27R was significantly elevated in plaques compared to control vessels, and co-localized to macrophages. (iii) *In vitro*, IL-27 increased NLRP3 inflammasome activation in monocytes with enhanced release of IL-1 β.

**Conclusions:**

We demonstrate increased levels of IL-27 and IL-27R in patients with carotid atherosclerosis. Our *in vitro* findings suggest an inflammatory role for IL-27, which can possibly be linked to atherosclerotic disease development.

## Introduction

Atherosclerosis is a progressive disorder in which lipids, immune cells and vascular cells accumulate in the arterial wall, leading to development of an atherosclerotic plaque. This lesion can rupture and cause adverse events like myocardial infarction (MI) and ischemic stroke, the most common causes of death in the world. Increased mechanistic insight is needed to improve treatment strategies for atherosclerotic diseases. As inflammatory activation is pivotal to the atherosclerotic process, a broader characterization of implicated inflammatory mediators is warranted [1–3].

Interleukin (IL)-27, a member of the IL-12 family of cytokines, is secreted as a heterodimer consisting of Epstein-Barr-induced gene 3 product (EBI3) and p28, with antigen presenting cells like macrophages and dendritic cells as its main cellular source. The IL-27 receptor (IL-27R) is also a heterodimer composed of IL-27RA and gp130, in which both receptor units are required for signal transduction. IL-27R is expressed on hematopoietic cells as well as by vascular endothelium and IL-27 has been shown to mediate both pro- and anti-inflammatory effects on various cell types [4]. Although most known for its regulation of T cells by inducing Th1 and suppressing Th17 differentiation, inflammatory effects are reported on monocytes and macrophages as well [5–8]. Altered expression of IL-27 is shown in autoimmune and infectious disorders, and therapeutic targeting of IL-27 is proposed in several of these [4, 9–11]. In atherosclerosis, increased expression of p28 and Ebi3 have been found in plaques, and elevated circulating IL-27 levels are reported in patients with coronary artery disease (CAD), associated with oxidized low-density lipoprotein (oxLDL) levels and disease severity [12–15]. Recently, two SNPs in the IL-27p28 gene were found to be associated with risk of premature CVD, however not associated with IL-27 plasma levels [16]. While the data from clinical studies may suggest that IL-27 has pro-atherogenic effects, IL-27 receptor knock out mice that are prone to develop atherosclerosis have increased atherosclerosis and inflammation [17, 18]. Furthermore, *in vitro* data illustrate anti-atherogenic effects of IL-27 on efflux of cholesterol from macrophages, whereas pro-atherogenic effects of IL-27 in endothelial activation and early plaque development have also been shown [19–21]. Together these findings point to a potential role for IL-27 in atherogenesis, but the mechanisms of action as well as the net effect of this cytokine in atherosclerosis is far from clear. Moreover, data on IL-27 expression in clinical atherosclerosis are scarce.

To further elucidate the role of IL-27 in atherosclerosis, we examined IL-27 levels in patients with carotid atherosclerosis, both systemically and within the lesion. Based on its role in macrophages/monocytes, we also examined the ability of IL-27 to modulate NLRP3 inflammasome activation, a recently reported pro-atherogenic mediator that mediates the activation of IL-1β.

## Materials and methods

### Ethics

The study protocols were approved by the Regional Health Authorities of South-Eastern and Western Norway. The study conforms to the principles outlined in the Declaration of Helsinki for use of human tissue or subjects. Signed informed consent was obtained from all participants.

### Blood samples from patients and control subjects

Patients (n = 140) with high-grade internal carotid stenosis (≥70%) treated with endarterectomy or carotid angioplasty with stenting were included in the study (Table 1). The patients were classified as asymptomatic (n = 85) or symptomatic (n = 55) in accordance to absence or presence of clinical symptoms (i.e., stroke, transient ischemic attack (TIA) or amaurosis fugax ipsilateral to the stenotic internal carotid artery within the past two months). Carotid stenoses were diagnosed and classified by precerebral color Duplex ultrasound and CT angiography according to consensus criteria [22, 23]. The asymptomatic carotid stenoses were detected during clinical examinations of patients with CAD, peripheral artery disease or stroke/TIA more than two months previously. Patients with concomitant inflammatory diseases, malignancies or overt liver and kidney disease were excluded. For comparison, plasma was collected from 19 sex- and age-matched apparently healthy subjects based on disease history and normal levels of C reactive protein (CRP). Blood samples from all patients and controls were drawn into pyrogen-free EDTA tubes, immediately placed on ice, and centrifuged at 2500*g* for 25 minutes to obtain platelet-poor plasma. Plasma was stored at -80°C until analyses.

**Table 1:** Baseline variables in patients from which plasma IL-27 is measured (n = 140).

	Patients N = 140
IL-27, (ng/mL)*	4.77 (0.01–40)
Age, (years)**	67.0 (8.5)
Male sex, %(n)	70.0 (98)
BMI, kg/m^2^ **	26.0 (3.7)
Smokers, % (n)	57.9 (81)
Hypertension, % (n)	65.7 (92)
Diabetes, % (n)	18.6 (26)
Aspirin treatment, % (n)	87.9 (123)
Statin treatment, % (n)	90.7 (127)
Degree of stenosis, %*	80 (60–99)
Echolucent plaque, % (n)	66.4 (93)
CRP, (mg/L)**	6.3 (8.3)
Leucocyte count, (10^9^/L)**	6.3(3.7)
Platelets, (10^9^/L)**	281.9 (74.1)
Total cholesterol, (mM)**	4.2 (1.0)
LDL cholesterol, (mM)**	2.6 (0.8)
HDL cholesterol, (mM)**	1.3 (0.4)
Triglycerides, (mM)**	1.5 (0.8)
HbA1c, (%)**	6.1 (1.4)

Numbers given as percentage (numbers) or * median (min-max) or ** mean (SD).

BMI: body mass index.

### Tissue sampling from carotid plaque and control tissue

Atherosclerotic carotid plaques (n = 169) were collected during carotid endarterectomy. Plaques that were used for RNA extraction were rapidly frozen in liquid nitrogen. Plaques that were used for immunohistochemistry (IHC) were placed in 4% phosphate buffered-formalin for 48 hours and then embedded in paraffin. For mRNA analyses, samples from the common iliac artery of organ donors were used as non-atherosclerotic vessel controls (n = 9). Control tissues were prepared and stored in the same way as carotid plaques. Only samples where all genes of interest were expressed are presented. For some of the genes outliers in the control group were identified by Grubbs test and removed from the respective analysis as described in figure legends.

### In vitro experiments

Cells from the human monocytic cell line THP-1 (American Type Culture Collection, Rockville, MD) were seeded in flat-bottomed microtitre plates (1.0–2.5x10^6^/mL; Costar, Sigma-Aldrich) in RPMI 1640 (PAA Laboratories, Pasching, Austria) supplemented with 10% fetal bovine serum (Gibco, Grand Island, NY) and 1% Penicillin-Streptomycin (5000U Penicillin, 5mg/mL Streptomycin, Sigma-Aldrich). The cells were stimulated with lipopolysaccharide (LPS, 10 ng/mL) from *Escherichia coli* (Sigma-Aldrich) for 6 hours, before adding IL-27 (100ng/mL, R&D Systems, Minneapolis, MN) over night. ATP (3mM, Sigma-Aldrich) was added one hour before cells and supernatants were harvested for protein and cell death analysis. For protein phosphorylation analyses the cells were stimulated with IL-27 (100 ng/mL) for 15 minutes in the absence of serum. In some experiments the NLRP3 inhibitor MCC950 (1μM) was added 30 minutes prior to ATP, as previously described [24]. For the gene expression analysis, IL-27 (5, 20, 50 or 100 ng/mL, R&D Systems), LPS (10 ng/mL) or a combination thereof were added to the cells for 3, 6 or 24 hours before they were harvested for RNA extraction.

Peripheral blood mononuclear cells (PBMCs) were obtained from buffy coats (The Blood Bank, Oslo University Hospital, Oslo) separated by gradient centrifugation using Lymphoprep (Nycomed, Oslo, Norway). PBMCs were seeded in microtitre plates (2x10^6^/mL; Costar, Sigma-Aldrich) in RPMI 1640 (PAA Laboratories) supplemented with 10% fetal bovine serum (Gibco) and incubated with IL-27 (100 ng/mL), IL-27 antibody (5 μg/mL, R&D Systems, Minneapolis, MN) or normal goat IgG control antibody (5 μg/mL, R&D Systems, Minneapolis, MN) overnight and then stimulated with LPS (10 ng/mL) for 6 hours. ATP (3 mM, Sigma-Aldrich) was added 30 minutes before supernatants were harvested for protein analysis.

Primary monocytes were isolated from PBMCs by plastic adherence. Cells were seeded in microtitre plates (10x10^6^/mL; Costar, Sigma-Aldrich) for one and a half hour before the media was removed and cells were washed twice. The adherent monocytes were then added RPMI 1640 (PAA Laboratories) supplemented with 10% fetal bovine serum (Gibco) and 1% Penicillin-Streptomycin (Sigma-Aldrich) and stimulated as PBMCs with IL-27, LPS and ATP before harvesting of supernatants for protein analysis.

### Supernatant analyses

Protein levels of IL-27 in plasma and IL-1β and TNF in cell supernatants were measured by enzyme immunoassays (EIAs) from R&D Systems. Quantification of lactate dehydrogenase was performed in fresh cell supernatants by Cytotoxicity Detection Kit from Sigma Aldrich (St. Louis, MO). Caspase-1 activity was assessed in fresh supernatants with Caspase-Glo 1 Inflammasome Assay from Promega (Madison, WI).

### Immunohistochemistry

Sections (5 μm) of paraffin-embedded atherosclerotic carotid plaques were treated with 0.5% H_2_O_2_, followed by high-temperature unmasking in citrate-buffer (pH 6.0), blocked with 0.5% bovine serum albumin (BSA) and then incubated with primary antibodies (anti-IL-27 and anti-IL-27RA; both goat-anti-human from Santa Cruz Biotechnology, San Diego, CA) for one hour at room temperature. After washing, the slides were incubated for an additional 30 minutes with peroxidase-conjugated secondary antibodies (anti-goat IgG, Impress-Vector, Vector laboratories, Burlingame, CA), rinsed and developed with chromogen for immunoperoxidase staining (DAB Plus, Vector laboratories) for seven minutes. The sections were counterstained with Hematoxylin. Omission of the primary antibody served as a negative control.

### Immunofluorescence

Paraffin-embedded sections (5 μm) of atherosclerotic carotid plaques were exposed to high-temperature unmasking (citrate-buffer, pH 6.0), blocked in 0.5% BSA and incubated over night at 4°C with primary antibodies (anti-IL-27, anti-IL-27RA; both goat-anti-human from Santa Cruz Biotechnology; and anti-CD68, mouse-anti-human, Dako, Glostrup, Denmark). The sections were counterstained with secondary antibodies, Alexa Fluor 488 and 568 (donkey-anti-goat and donkey-anti-mouse, respectively, from Invitrogen, Eugene, OR). Nuclei were stained with diamidino-2-phenylindole (DAPI; Slow Fade Gold antifade reagent, Invitrogen). Fluorescent images were obtained on a Nikon Eclipse E400 microscope with 400× magnification.

### Western blotting

Proteins were extracted from THP-1 monocytes stimulated with LPS and IL-27 as described. In brief, cells were washed with PBS and lysed in Mammalian Protein Extraction Reagent (M-PER) containing HALT protease and phosphatase inhibitor cocktail (ThermoFisher Scientific, Waltham, MA). The samples were denatured in 5x Loading buffer (ThermoFisher Scientific), separated on a SDS-PAGE gel (Bio-Rad) in SDS Running buffer (Bio-Rad) and transferred to a PVDF transfer membrane (ThermoFisher Scientific). Blotting was performed using 20% methanol, 25mM Tris base and 192mM glycine pH 8.3 (Bio-Rad). The membranes were blocked in Superblock (ThermoFisher Scientific) or 5% skimmed milk/bovine serum albumin (BSA) in Tris-buffered saline containing 0.1% Tween-20 (TBS-T) for one hour at room temperature and then incubated with primary antibodies for IL-1β (1:2000, R&D Systems) in 20% Superblock (ThermoFisher Scientific), NLRP3 (1:1000, Cell Signaling, Denvers, MA), β-tubulin (1 μg/ml, Sigma Aldrich) STAT1 and STAT3 (1:1000, Cell Signaling) in 5% skimmed milk, phosphorylated (p)STAT1, pSTAT3, pIκB and total IκB (1:1000, Cell Signaling) in 5% BSA at 4°C overnight. Membranes were then washed with TBST and incubated with species-specific horseradish peroxidase-conjugated secondary antibody in 20% Superblock or 5% skimmed milk. The membrane were incubated with chemiluminescent substrate (SuperSignal West Dura, ThermoFisher Scientific) and visualized by a CCD-camera based digital imaging system (LAS-4000).

### Real-time quantitative RT-PCR

Total RNA was obtained from atherosclerotic and non-atherosclerotic vessels and from monocytes with the use of RNeasy spin columns (QIAGEN, Hilden, Germany). All samples were subjected to DNase treatment (RNase-Free DNase Set; QIAGEN) and stored at -80°C until further analysis. cDNA synthesis was performed using the High-Capacity cDNA Reverse Transcriptation Kit (Applied Biosystems, Foster City, CA). Gene expression was examined by real-time quantitative (q)PCR. mRNA detection of NLRP3, IL-1β, MD-2, TLR4, CD14, CD39 and reference genes GAPDH and β-actin was assessed with SybrGreen primers (Applied Biosystems): (NLRP3, Forward primer (FP): AGCTTCAGGTGTTGGAATTAGACA, Reverse primer (RP): GCTGGAGGTCAGAAGTGTGGA; IL-1β, FP: ATGATGGCTTATTACAGTGGCAA, RP: GTCGGAGATTCGTAGCTGGA; MD2, FP: CCACCCTGTTTTCTTCCATATTTAC, RP: ATTGCATTTTATCACAGTAGGTGTATGA; TLR4, FP: GCTGGATTTATCCAGGTGTGAA, RP: AAAAGGCTCCCAGGGCTAA; CD14, FP: ACGCCAGAACCTTGTGAGC, RP: GCATGGATCTCCACCTCTACTG; CD39, FP: AGCAGCTGAAATATGCTGGC, RP: GAGACAGTATCTGCCGAAGTCC; GAPDH, FP: GCCCCCGGTTTCTATAAATTG, RP: GTCGAACAGGAGGAGCAGAGA; β-actin, FP: AGGCACCAGGGCGTGAT, RP: TCGTCCCAGTTGGTGACGAT). Sequence specific TaqMan primers and probes were used for detection of IL-27p28, IL-27RA and Ebi3 mRNA (Assay-ID IL-27p28: Hs00377366_m1; IL-27RA: Hs00945029_m1; Ebi3: Hs01057148_m1 and, Applied Biosystems). The relative mRNA level of each transcript was calculated by the ΔΔCt-method and normalized to controls (control tissue, unstimulated- or LPS-stimulated cells).

### Statistical analysis

The Mann-Whitney U test was used to determine statistical differences between patients and controls. Coefficient of correlation was calculated by the Spearman rank test. For cell culture experiments, Student`s t-test, one- or two-way ANOVA was used as appropriate. Statistical analyses were performed using Prism version 6.0 (GraphPad software, La Jolla, CA) and statistical significance was considered at p<0.05.

## Results

### Increased circulating levels of IL-27 in patients with carotid atherosclerotic disease

**Fig 1:**
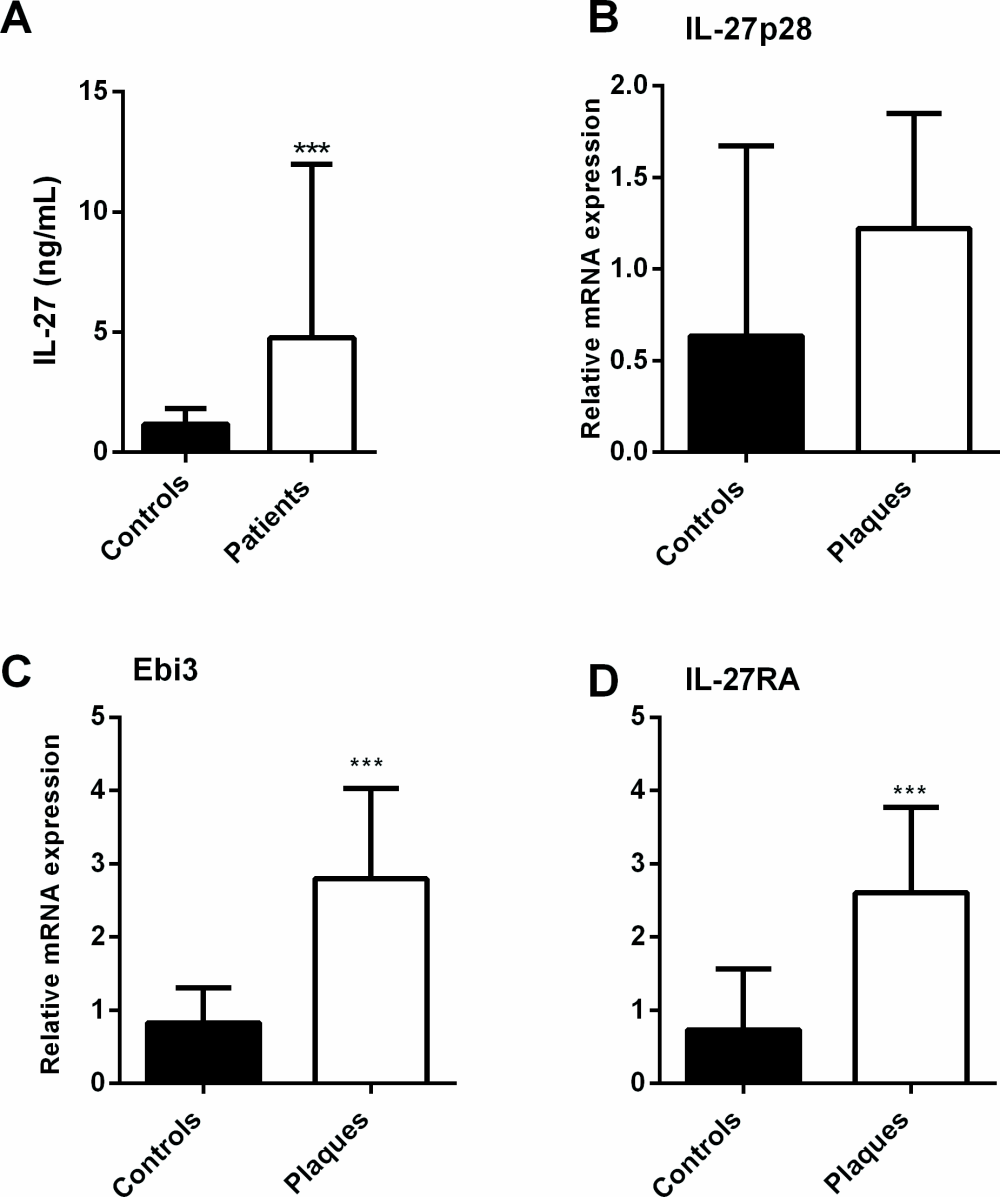
Increased plasma levels of IL-27 and plaque gene expression of IL-27 and IL-27R in patients with carotid atherosclerosis. Plasma levels of IL-27 (**A**) were measured with EIA in plasma from patients with asymptomatic (n = 85) and symptomatic carotid atherosclerosis (n = 55) and in healthy controls (n = 19). Gene expression of IL-27 subunits IL-27p28 (**B**) and Ebi3 (**C**) and the IL-27RA (**D**) were measured by qPCR in carotid plaques (n = 159) and in healthy controls (n = 9). Expression was related to the mean of the reference genes β-actin and GAPDH and normalized to control samples. Data are presented as median and interquartile range. ***p<0.001 versus controls.

Plasma levels of IL-27 were significantly raised in patients with carotid atherosclerosis (n = 140) as compared with healthy controls (n = 19) (median [interquartile range]: 4.77 [1.82–11.99] ng/mL versus 1.17 [0.85–1.80] ng/mL, p<0.001, Fig 1A). We found the same pattern, with raised IL-27 levels in patients with asymptomatic (n = 85) and symptomatic (n = 55) disease (i.e., patients with no symptoms or symptoms more than two months ago versus those with symptoms within the last two months, respectively) compared to controls, without any statistical difference between the two groups of patients (p = 0.91).

### Increased expression of IL-27 and IL-27R in carotid atherosclerotic plaques

**Fig 2:**
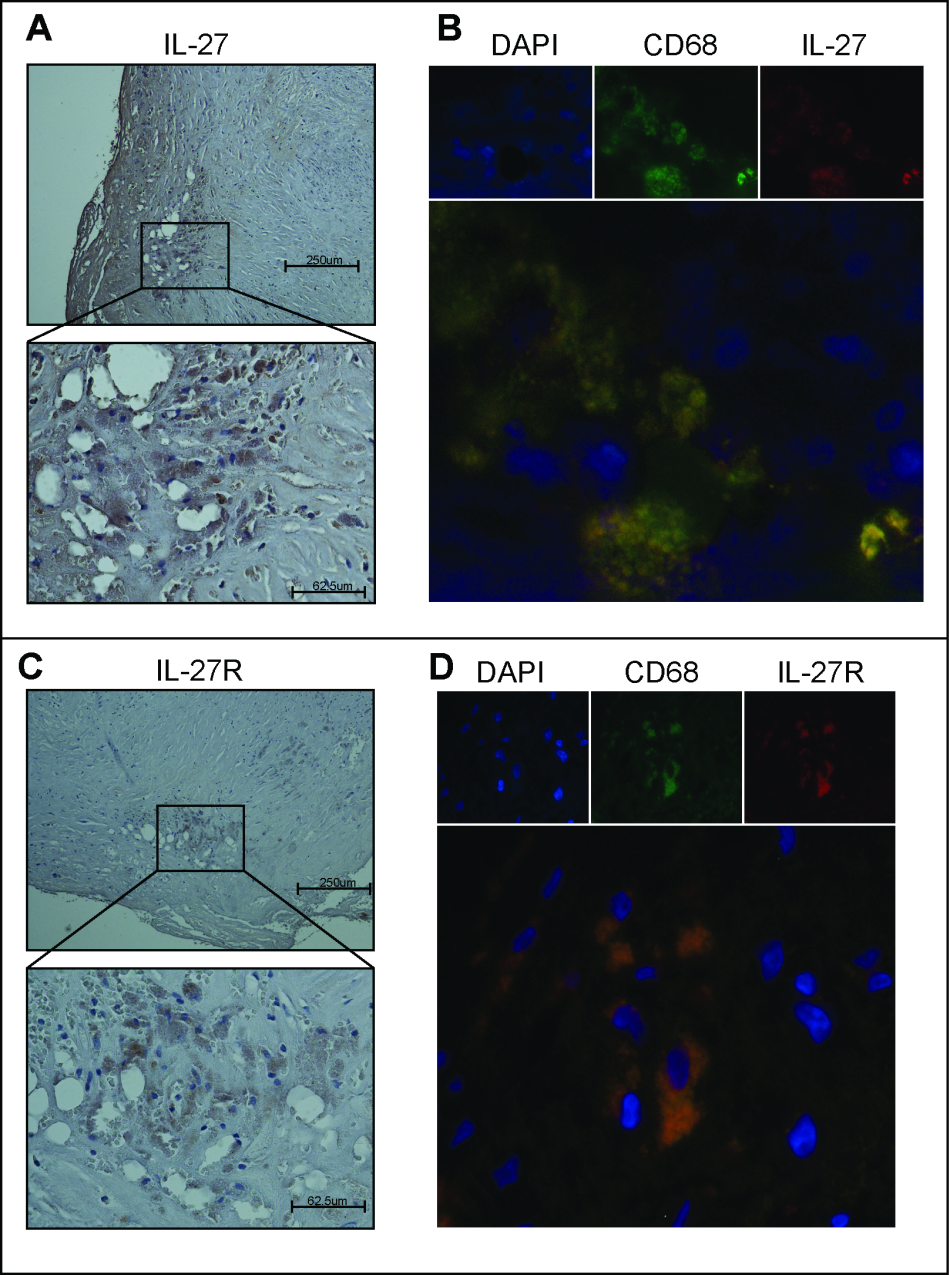
Protein expression of IL-27 and IL-27R in carotid plaques are co-localized to macrophages. The two left panels show immunostaining of IL-27 (**A**) and IL-27R (**C**) in symptomatic carotid plaques. Images obtained with 100x (top) and 400x (bottom) magnification. The two right panels show double immunofluorescent staining of IL-27 (**B**) and IL-27R (**D)**. IL-27 and IL27R are shown by red fluorescence, CD68+ macrophages by green fluorescence and nucleus (DAPI) by blue fluorescence. The bottom right picture is a merge of IL-27, CD68 and DAPI or IL-27R, CD68 and DAPI, respectively. The figure shows data representative of six symptomatic patients.

qPCR analyses showed increased levels of IL-27p28 (p = 0.06), Ebi3 (p<0.001) and IL-27RA (p<0.001) mRNA in carotid plaques (n = 159) compared to control samples (n = 9), although the difference for IL-27p28 did not reach statistical significance (Fig 1B–1D). At the protein level, IL-27 and IL-27R protein expression within the lesion was confirmed by immunohistochemistry and co-localized to CD68^+^ macrophages (Fig 2).

### IL-27 induces NLRP3 dependent release of IL-1β from monocytes

**Fig 3:**
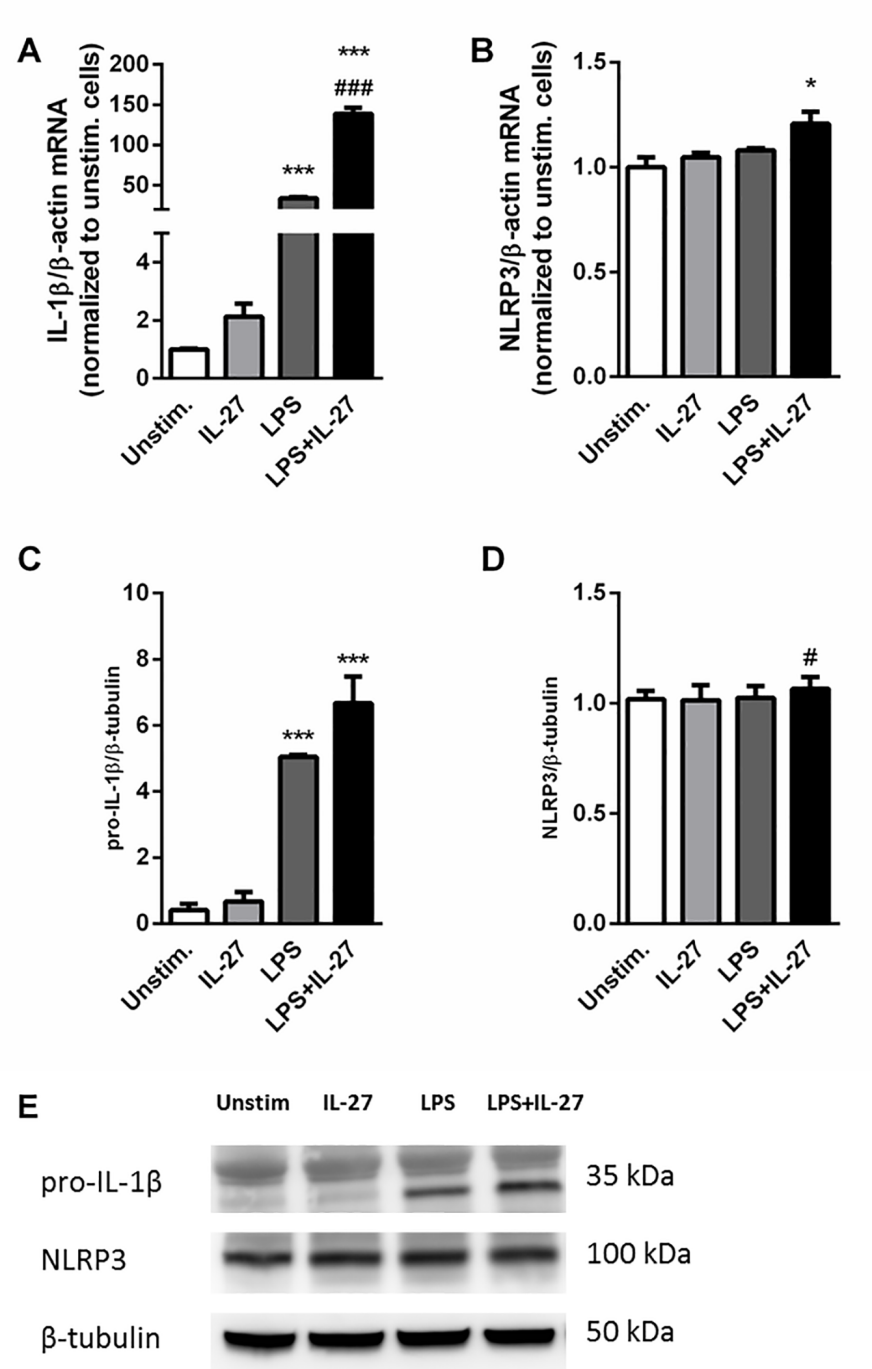
IL-27 increases LPS-induced IL-1β expression in THP-1 cells. Gene expression analyses of IL-1β (**A**) and NLRP3 (**B**), THP-1 cells were stimulated with LPS (10 ng/mL) and/or IL-27 (100 ng/mL) for 24 hours. Gene expression was analyzed by qPCR, related to the reference gene β-actin and normalized to unstimulated cells. Results are representative of a minimum of three experiments and data are presented as mean and SEM. For protein analyses of pro-IL-1β (**C** and **E**) and NLRP3 (**D** and **E**), THP-1 cells were primed with LPS (10 ng/mL, 6 hours) and incubated over night with IL-27 (100 ng/mL). Pro-IL-1β, NLRP3 and β-tubulin protein expression were analyzed by western blotting in protein homogenates from cells. Data are presented as mean and SEM of six (pro-IL-1β) and three (NLRP3) independent experiments and representative Western blots. *p<0.05 and ***p<0.001 versus unstimulated cells, #p<0.05 and ### p<0.001 versus LPS-stimulated cells.

**Fig 4:**
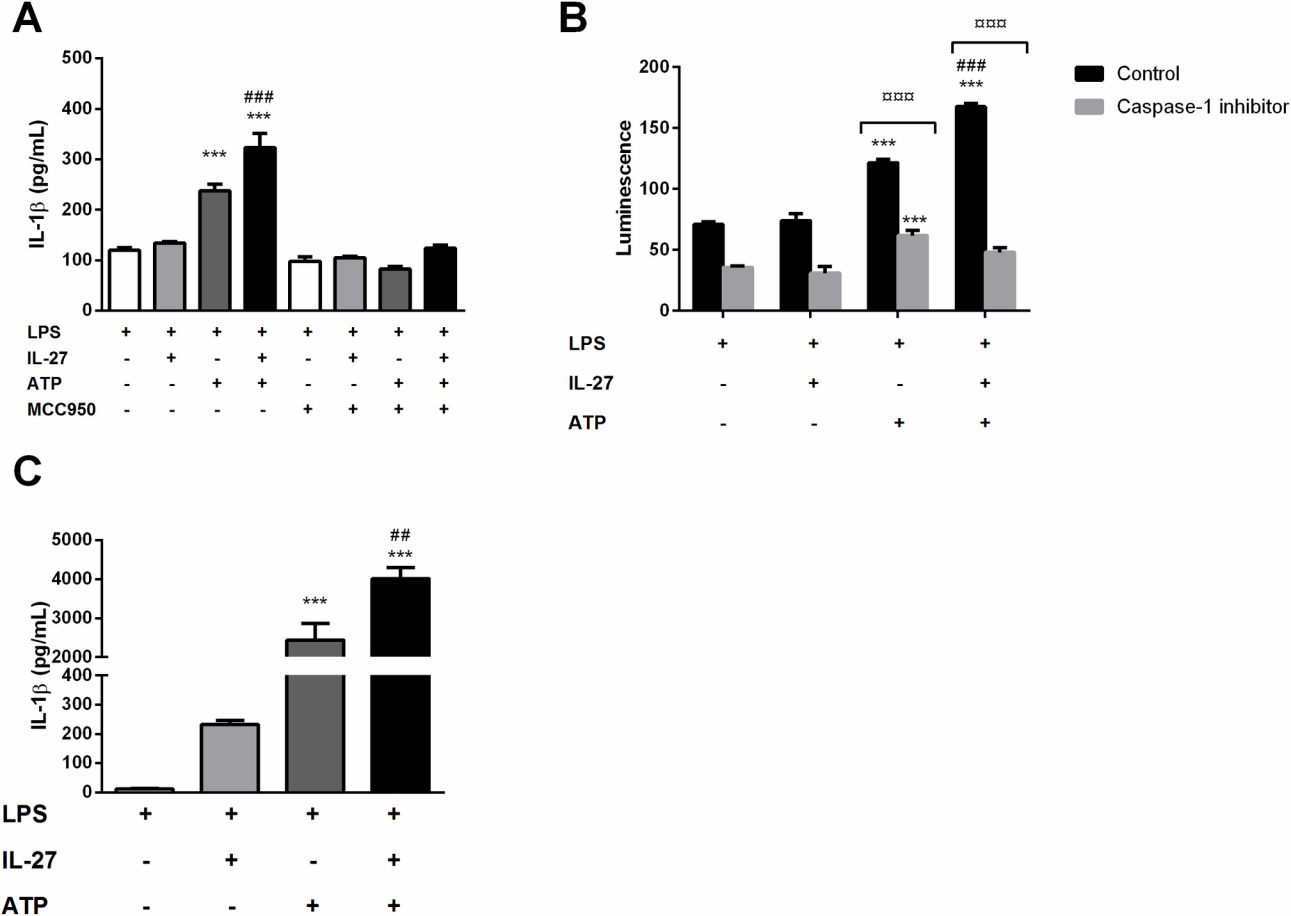
IL-27 induces NLRP3 dependent release of IL-1β from monocytes. THP-1 cells were primed with LPS (10 ng/mL, 6 hours) and incubated over night with IL-27 (100 ng/mL) before stimulation with ATP (3 mM, 1 hour). The NLRP3 inhibitor MCC950 (1μM) was added 30 minutes prior to ATP as indicated. Primary monocytes were incubated with IL-27 over night (100 ng/mL) before stimulation with LPS (10 ng/mL) for 6 hours and ATP (3 mM) for 30 minutes. IL-1β was measured in supernatants from THP-1 cells (**A**) and primary monocytes (**C**) with EIA. (**B**) Caspase-1 activity was measured by bioluminescent luciferase activity in fresh cell-free supernatants from THP-1 cells stimulated as described above, with or without the presence of Ac-YVAD-CHO caspase-1 inhibitor after the manufacturer’s instructions. Results are representative of minimum three experiments and data are presented as mean and SEM. Only LPS-primed samples are shown. ***p<0.001 LPS-stimulated cells, ###p<0.001 versus LPS/ATP-stimulated cells and ¤¤¤p<0.001 versus control cells without caspase-1 inhibitor (B).

IL-27 is known to have both pro- and anti-inflammatory properties, with effects on monocytes and macrophages in addition to its more described effects on T cells. The effect of IL-27 on NLRP3 inflammasome activation in monocytes, a pro-inflammatory mediator of atherosclerosis [25, 26], was also very recently described [27]. The NLRP3 inflammasome is activated in a two-step manner. A priming signal, signal 1, leads to transcription of IL-1β and NLRP3 mRNA, and the activation signal, signal 2, leads to assembly of the NLRP3 inflammasome complex. This activates caspase-1, that again cleaves the inactive pro-IL-1β into its active form, which is then subsequently released [28]. We stimulated THP-1 cells with LPS (a well-known signal 1) in combination with IL-27 (100 ng/mL) and measured gene expression of IL-1β as well as protein expression of pro-IL-1β. At 24 hours, IL-27 alone had no effect on IL-1β mRNA levels but markedly enhanced LPS-induced IL-1β gene expression (Fig 3A). On pro-IL-1β protein expression, however, IL-27 only showed a moderate, non-significant increase of LPS-induced expression (Fig 3B and 3E). Notably, the THP-1 cells seemed to have a high basal level of NLRP3, and stimulation with LPS or IL-27 alone had no effect on NLRP3 expression at mRNA level. The combination of IL-27 and LPS stimulation gave a small, however significant increase in NLRP3 gene expression (Fig 3B). Similar, on the protein level IL-27 stimulation gave a small, significant increase in LPS-induced NLRP3 expression, however these levels were not significantly increased compared to unstimulated cells (Fig 3D and 3E). To study the effect of IL-27 on IL-1β release, we added ATP (a well-known signal 2). As expected, stimulation with ATP induced IL-1β release from cells primed with LPS, and IL-27 significantly increased the LPS/ATP-mediated IL-1β release (Fig 4A). Moreover, NLRP3 inflammasome inhibition by MCC950 significantly reduced the IL-27-induced effect on IL-1β release, clearly indicating the dependence of NLRP3 (Fig 4A). Moreover, addition of IL-27 to LPS and ATP-treated cells significantly increased caspase-1 activity further supporting a role for IL-27 in NLRP3 inflammasome activation, (Fig 4B). The effect of IL-27 was confirmed in human primary monocytes, were IL-27 significantly increased LPS/ATP -induced IL-1β release (Fig 4C).

Thus, IL-27 enhanced the LPS induced mRNA levels, however had rather modest effects on pro-IL-1β, suggesting that IL-27 in addition to increasing IL-1β mRNA levels, at least partly promote NLRP3 inflammasome activation by enhancing signal 2. The IL-27 induced release of IL-1β was not a result of increased cell death, as measured by lactate dehydrogenase activity in cell supernatants (S1 Fig).

### Dose-dependent effects of IL-27 stimulation on IL-1β release and CD39 expression in human monocytes

**Fig 5:**
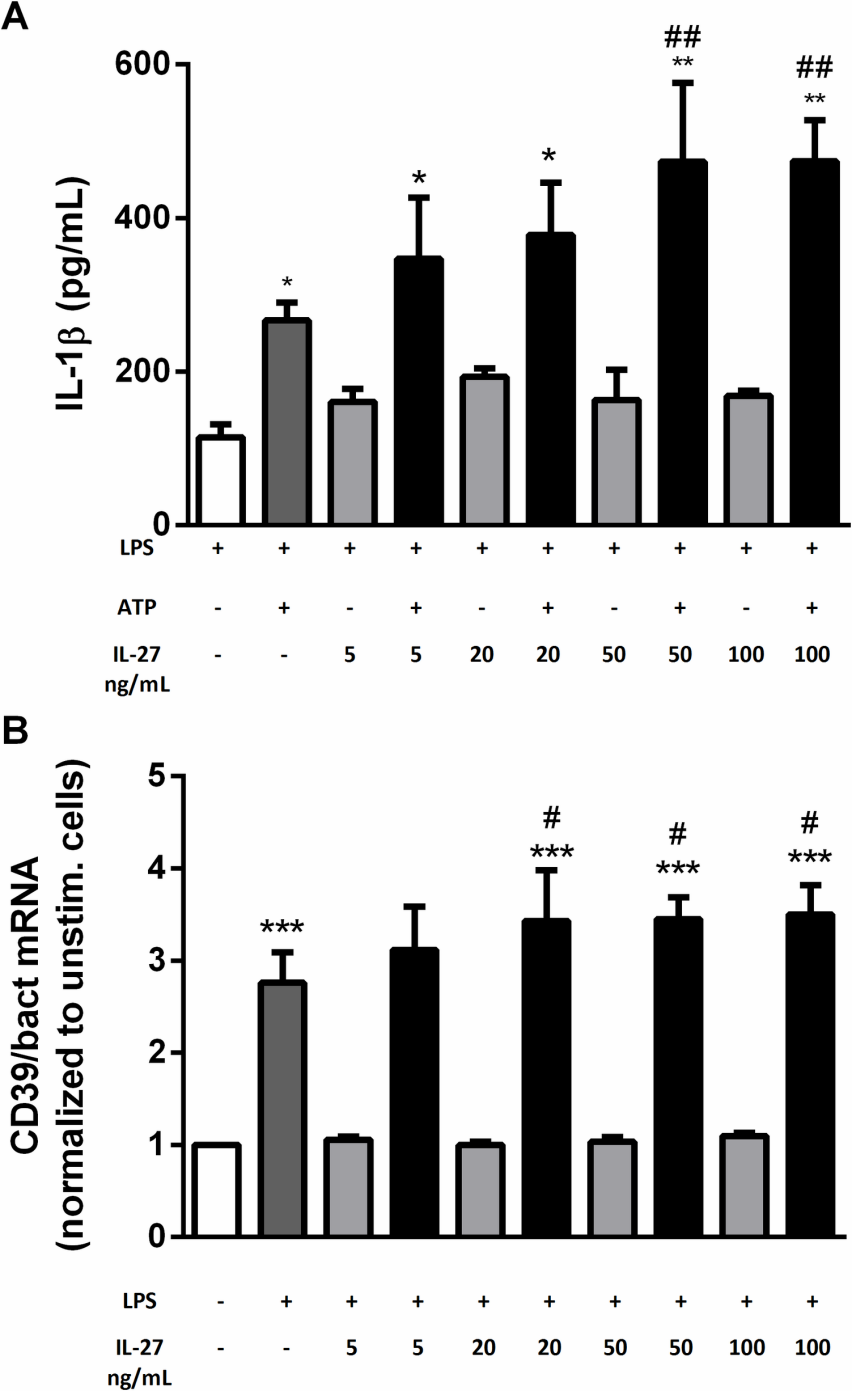
Dose-dependent effects of IL-27 on IL-1β release and CD39 expression in human monocytes. **A)** THP-1 cells were primed with LPS (10 ng/mL, 6 hours) and incubated over night with IL-27 (5, 20, 50 and 100 ng/mL) before stimulation with ATP (3 mM, 1 hour). IL-1β release was measured in supernatants with EIA. Data are presented as mean and SEM of three separate experiments. Only LPS-stimulated samples are shown. *p<0.05 versus LPS-stimulated cells, **p<0.01 versus LPS-stimulated cells and ##p<0.01 versus LPS/ATP-stimulated cells. **B)** THP-1 cells were stimulated with LPS (10 ng/mL) and/or IL-27 (5, 20, 50 and 100ng/mL) for 24 hours. Gene expression analyses of CD39 was done by qPCR, related to the reference gene β-actin and normalized to unstimulated cells (**B**). Data are presented as mean and SEM of four separate experiments. ***p<0.001 versus unstimulated cells (white bar), and #p<0.05 versus LPS-stimulated cells.

Mascanfroni et al. have shown that low doses of IL-27 (20 ng/mL) inhibit NLRP3 inflammasome activation in mouse dendritic cells, through induction of the endonuclease CD39 [29]. In contrast, we found a dose-dependent increase in IL-27-mediated IL-1βrelease in LPS/ATP exposed THP-1 monocytes. However only high doses of IL-27 (50 and 100 ng/mL) resulted in significant IL-1β release when all experiments were combined (Fig 5A). Moreover, IL-27 stimulation resulted in a parallel, modest increase in LPS-induced CD39 mRNA levels (Fig 5B). This may suggest that the interplay between IL-27, CD39 and the NLRP3 inflammasome has a different impact in human monocytes, than as previously demonstrated in mouse dendritic cells [29].

### IL-27 facilitates LPS signaling in THP-1 monocytes

**Fig 6:**
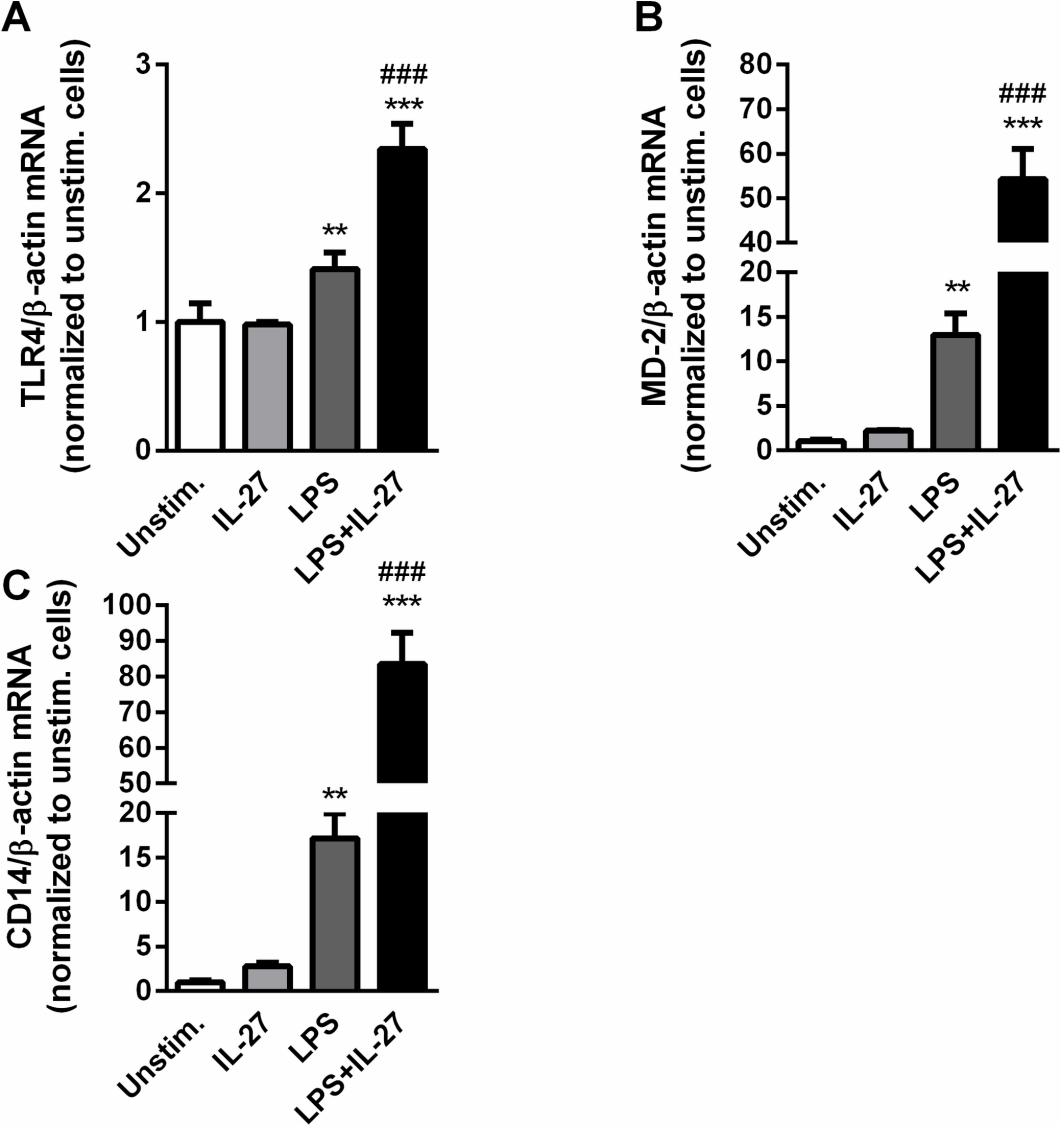
IL-27 enhances LPS-induced gene expression in THP-1 cells. THP-1 cells were stimulated with LPS (10 ng/mL), IL-27 (100 ng/mL) or a combination thereof for 24 hours. Gene expression analyses were done by qPCR, related to reference gene β-actin and normalized to unstimulated cells. The figure shows mRNA levels of TLR4 (**A**), MD-2 (**B**) and CD14 (**C**). Results are representatives of minimum three experiments and data are presented as mean and SEM. **p<0.01 and ***p<0.001 versus un-stimulated cells, ###p<0.001 versus LPS-stimulated cells.

Our findings so far have shown that IL-27 stimulation can boost the effect of LPS on IL-1β mRNA levels in THP-1 monocytes. LPS signals through TLR4, facilitated by LPS binding protein (LBP), CD14 and MD-2. In an ordered reaction pathway these proteins alter the physical presentation of endotoxin, resulting in amplification of host responsiveness to LPS through TLR4 [30]. It has previously been demonstrated that IL-27 enhances TLR4 expression in monocytes, and localization of CD14 to TLR4 [7], and it was recently shown that IL-27 gave a modest increase in MD-2 expression in CD14^+^ THP-1 cells [27]. The effect of IL-27 on LBP is, however, to the best of our knowledge, unknown. To further examine the interaction between LPS and IL-27, mRNA levels of TLR4, CD14 and MD-2 were measured in THP-1 monocytes that were stimulated with IL-27, LPS or a combination thereof for 3, 6 and 24 hours. As for IL-1β gene expression, the clearest responses were seen after 24 hours, and are presented here. LPS induced a significant increase in all these genes and IL-27 markedly boosted the LPS-stimulated effect, with a particularly enhancing effect on CD14 and MD-2 (Fig 6). These findings further underscore the ability of IL-27 to facilitate LPS-mediated effects in monocytes, possibly by up-regulation of the receptors/co-receptors for LPS-signaling.

### IL-27 signals through STAT pathways

Our results suggest that IL-27 potentially enhances the LPS-induced production of inflammatory cytokines from monocytes through increased expression of TLR4, CD14 and MD-2. Guzzo et al. have previously shown that this occurs in a STAT3-NF-kB-dependent manner and that IL-27 signal through STAT1, STAT3 and NF-kB activation in human monocytes and macrophages [7]. We confirm herein that IL-27 induces phosphorylation of STAT1 and STAT3, however not IκB, in THP-1 cells. The lack of IL-27-induced IkB phosphorylation could, at least partly, reflect a high degree of phosphorylation in cells without IL-27 exposure (S3 Fig). These findings may suggest that STAT1 and STAT3 could be involved in the demonstrated effects of IL-27 on NLRP3 activation in THP-1 monocytes. However, owing to technical reasons/unreliable results by using specific signaling blockers, we were unfortunately not able to more precisely demonstrate the role of these transcriptional factors in the IL-27 induced release of IL-1β in LPS/ATP exposed THP-1 cells.

### IL-27 promotes inflammasome dependent and independent inflammatory responses in PBMCs

**Fig 7:**
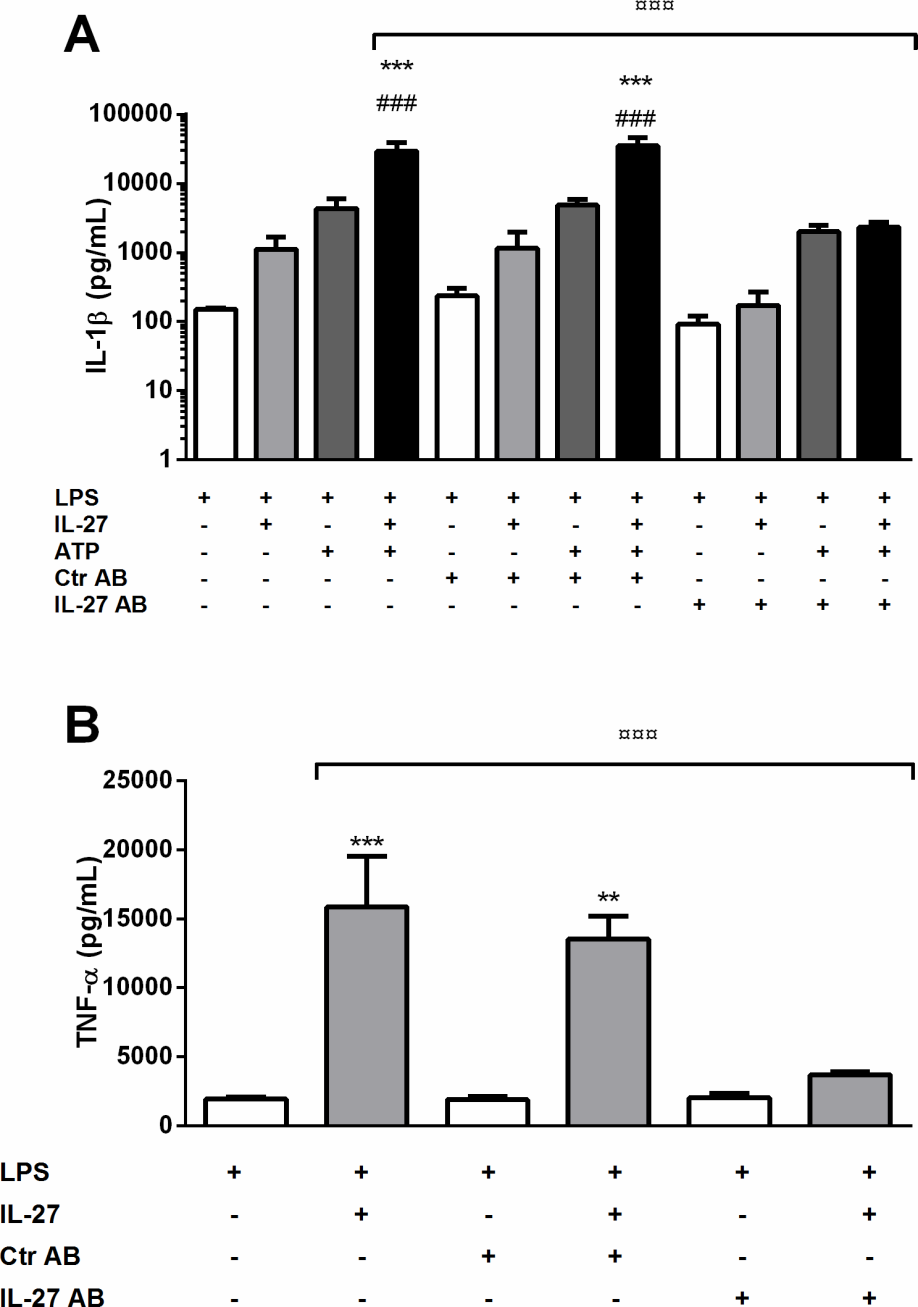
IL-27 increases IL-1β and TNF release from PBMCs. PBMCs were incubated with IL-27 (100 ng/mL) with our without IL-27 antibody or control antibody (5 μg/mL) over night before stimulation with LPS (10 ng/mL) for 6 hours and ATP (3 mM) for 30 minutes. IL-1β (**A**) and TNF (**B**) release was measured in supernatants with EIA. Results are presented as mean and SEM of four (IL-1β) and five (TNF) separate experiments. **p<0.01 and ***p<0.001 versus LPS-stimulated cells, ###p<0.001 versus ATP/LPS-stimulated cells, ¤¤¤p<0.001 versus LPS/ATP/IL-27-stimulated cells (IL-1β) and LPS/IL-27-stimulated cells (TNF).

To further explore the pro-inflammatory effects of IL-27, we exposed PBMCs from healthy blood donors to IL-27 and LPS/ATP (inflammasome activation) as well as to IL-27/LPS without ATP (inflammasome independent activation). IL-27 significantly enhanced the LPS/ATP-induced IL-1β release from PBMCs (Fig 7A). IL-27 also significantly increased the LPS-induced release of TNF from these cells (Fig 7B), demonstrating that IL-27 exerts both inflammasome dependent and independent inflammatory effects in PBMCs.

### LPS does not induce IL-27 release in PBMCs

Previous studies have shown that monocytes release IL-27 after LPS-stimulation and that IL-27 can enhance LPS-induced inflammatory effects, suggesting a positive feed-forward loop in these cells [7, 31]. To elucidate the possibility of such a mechanism in PBMCs, we measured IL-27 release from PBMCs after stimulation with LPS alone or in combination with ATP. The PBMCs did however not release IL-27 in response to either LPS or LPS/ATP activation. The IL-27 levels released from the PBMCs were, however, in general very low and barely detectable by the assay, which could influence on the results (S2 Fig). Nonetheless, inhibition of IL-27 did not reduce LPS-induced levels of IL-1β or TNF (Fig 7A and 7B) further arguing against a feed-forward interaction between LPS and IL-27 in these cells. The PBMC pool is however a heterogeneous mix of cells, which could mask the effects of individual cell populations. On the other side, PBMCs are more comparable to the *in vivo* situation.

### Upregulation of NLRP3 inflammasome components in carotid atherosclerotic plaques

**Fig 8:**
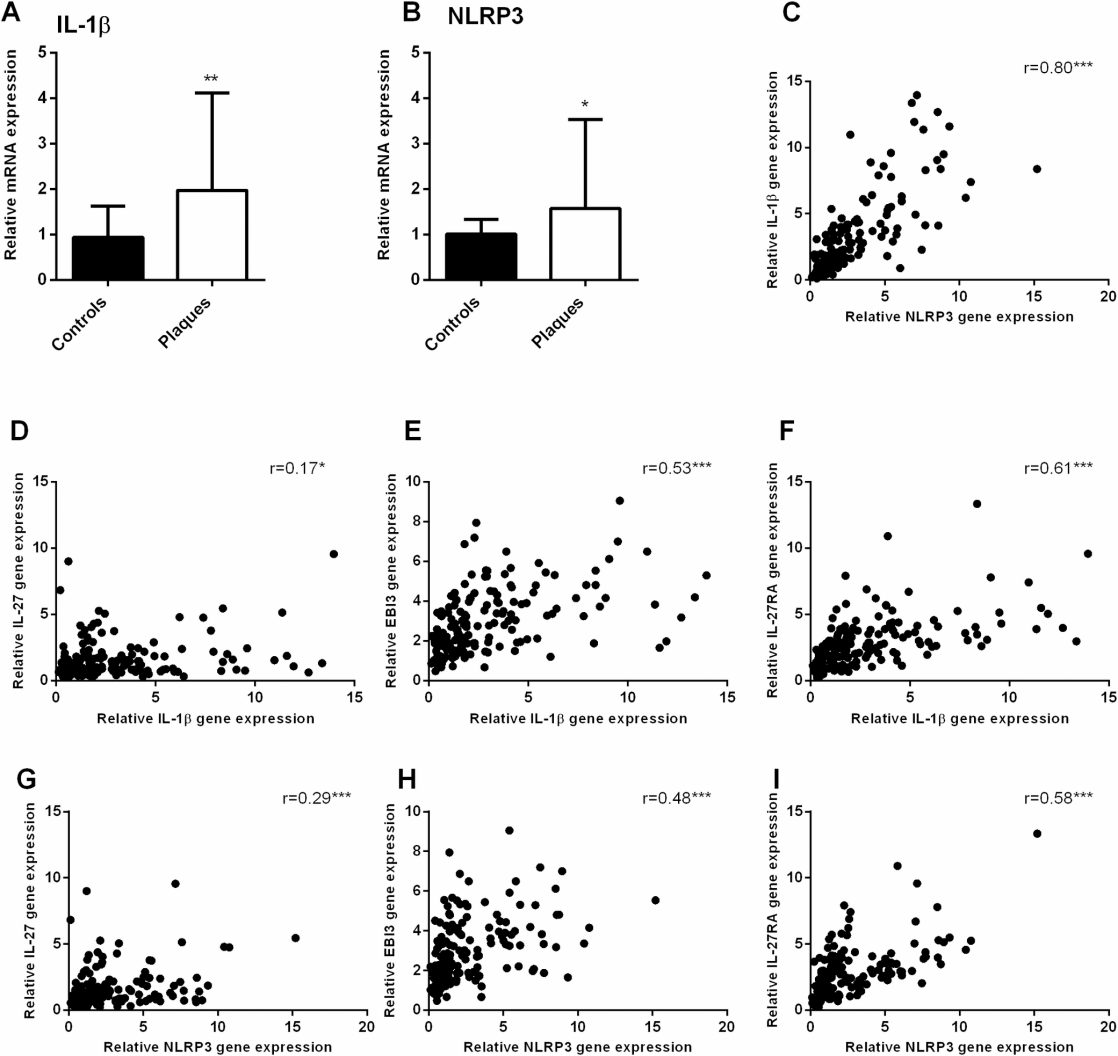
Increased gene expression of IL-1β and NLRP3, and positive correlations to IL-27, Ebi3 and IL-27RA in human carotid plaques. Gene expression of IL-1β (**A**) and NLRP3 (**B**) were measured by qPCR in carotid plaques (n = 159) and in healthy controls (n = 9). For NLRP3 one, and for IL-1β two control sample were identified as outliers and excluded, leaving n = 8 and n = 7 control samples, respectively. Expression was related to the mean of the reference genes β-actin and GAPDH and normalized to control samples. Data are presented as median and interquartile range. Correlations (**C-I**) were calculated by the Spearman’s rank correlation test and are presented by r and p values, *p<0.05, *p<0.01 and ***p<0.001.

We have previously demonstrated increased expression of NLRP3-related genes in carotid artery plaque tissue [26]. Here we confirm these findings in a different, larger cohort, showing increased expression of IL-1β and NLRP3 expression in carotid plaques compared to healthy control arteries (Fig 8A and 8B). To further explore a possible interplay between the IL-27 and NLRP3 system, we performed correlation analyses between the expression of IL-27p28, IL-27RA, Ebi3 and IL-1β and NLRP3. IL-27p28 (r = 0.17, p = 0.04 and r = 0.29, p<0.01), Ebi3 (r = 0.53, p<0.01 and r = 0.48, p<0.01) and IL-27RA (r = 0.61, p<0.01 and r = 0.58, p<0.01) were all positively correlated to IL-1β and NLRP3 expression, respectively, although some of the correlations were rather modest. As expected, expression of IL-1β and NLRP3 in the plaques had a strong positive correlation (r = 0.80, p<0.01). These analyses further support the possibility of interplay between these two systems, not only *in vitro* but also *in vivo* (Fig 8C–8I).

## Discussion

Herein we show an up-regulation of the IL-27 system in patients with carotid atherosclerosis both systemically (IL-27) and within the atherosclerotic lesion (IL-27 and IL-27R). Moreover, our *in vitro* experiments show the ability of IL-27 to enhance inflammasome activation in monocytes and PBMCs by facilitating the priming effects of LPS (signal 1) and enhancing the effect of ATP (signal 2). Our findings may suggest a role for IL-27 in clinical atherosclerosis, at least partly through its enhancing effects on LPS-mediated functions and NLRP3 inflammasome activation. The inflammatory effects of IL-27 in these cells, seem also however to be mediated through NLRP3-independent mechanisms (i.e., TNF release).

Others have previously shown an alteration in IL-27 levels in coronary atherosclerosis [12–14, 16], and expression of the IL-27 subunits Ebi3 and p28 in carotid plaques is previously described in a small patient cohort [15]. Herein we extend these reports by demonstrating increased plasma levels of IL-27 in 140 patients with carotid atherosclerosis, and increased mRNA levels of IL-27 subunits and IL-27RA, as well as increased expression of IL-1β and NLRP3 within the carotid lesion from 159 patients. Although the effect of IL-27 in atherosclerosis is still unknown, our findings herein, showing enhanced expression of both the IL-27 system and components of the NLRP3 inflammasome within the carotid plaque and NLRP3-dependent inflammatory effects of IL-27 in PBMCs and monocytes, may suggest an inflammatory effect of IL-27 in clinical atherosclerosis.

The NLRP3 inflammasome is implicated in several inflammatory conditions and also in atherosclerotic disease. In host defense, NLRP3 inflammasome activation gives protection from invading microbes, but if dysregulated, this activation can lead to harmful inflammatory conditions[25, 26, 32–35]. NLRP3 inflammasome activation is therefore carefully regulated through a two-step activation process with a priming signal increasing transcription of IL-1β and NLRP3, and a second signal leading to complex assembly and cleavage of pro-caspase-1 and IL-1β release [28]. However, the molecular mechanisms behind this activation are not completely understood. IL-27 has recently been shown to increase LPS-induced release of IL-1β from human THP-1 cells, primary human monocytes and murine monocytes in an NLRP3 dependent fashion [27]. In the present study, we extend these findings, by showing that IL-27 contributes to NLRP3 activation also in human PBMCs. Further, we show that IL-27 promotes inflammatory responses in these cells in both a NLRP3 inflammasome-dependent and independent manner. IL-27 stimulation markedly boosted the LPS-stimulated expression of IL-β. More importantly, IL-27 significantly increased LPS/ATP-induced caspase-1 activity and NLRP3-dependent IL-1β release from monocytes and PBMCs. The priming effect of IL-27 on LPS-induced IL-1β mRNA may suggest that IL-27 stimulates NLRP3 inflammasome activation by enhancing LPS-mediated effects (signal 1). However, IL-27 had only modest and non-significant effects on LPS-induced pro-IL-β levels, suggesting that IL-27 at least partly promotes IL-1β by enhancing the effects of ATP (signal 2). This is also supported by the IL-27 induced caspase-1 activity showed recently by others [27], and herein. It has previously been shown that IL-27 can increase the LPS response in monocytes, in a STAT3-NF-κB-dependent manner, through induced expression of TLR4 and CD14-TLR4 relocalization [7]. Here we found that IL-27 also increased LPS-induced MD-2 and CD14 expression, strengthening the role for IL-27 in LPS-mediated signaling. Further we demonstrate that IL-27 amplify LPS/ATP-induced IL-1β and LPS-induced TNF release from PBMCs, demonstrating that IL-27 can potentiate LPS-induced inflammation in both an NLRP3 inflammasome-dependent and independent manner.

Our *in vitro* studies in monocytes and PBMCs may not necessarily reflect the *in vivo* situation within the carotid lesion. However, the up-regulation of IL-27 and its receptor, co-localized to macrophages and correlated to the expression of NLRP3 inflammasome components within the carotid plaques, may suggest that this interplay also is operating *in vivo*. In support of this, both others and we have demonstrated enhanced NLRP3 inflammasome activation in carotid atherosclerosis [26, 32]. Moreover, ATP is released during cell necrosis[36], which clearly could be relevant within an atherosclerotic lesion, particularly during plaque destabilization. Also, although inflammatory cytokines like TNF could “replace” LPS as signal 1, endotoxins are also relevant for atherogenesis, and endotoxemia is shown to be a strong risk factor for atherosclerosis [28, 37, 38]. In patients with coronary atherosclerosis, increased levels of both IL-27 and NLRP3 inflammasome components are reported and are also associated with disease severity [13, 33]. Although *in vivo* confirmation is needed, our results suggest that IL-27 could interact with the NLRP3 inflammasome also in patients with carotid atherosclerosis potentially contributing to plaque progression.

Atherosclerotic models in mice have shown possible anti-atherogenic effects of IL-27 signaling, and anti-inflammatory effects of IL-27 on macrophages [17, 18]. However, the contrast to our findings can be due to the pleiotropic effects of IL-27 [4]. IL-27 has previously been shown to have both pro- and anti-inflammatory effects, and to alter the same mediators in different directions depending on co-stimuli and cell type [7, 8]. Therefore, the local environment seems important for the effects of IL-27. Moreover, total loss of IL-27 signaling as in IL-27RA deficient mice, may not necessarily mirror the situation in patients with carotid atherosclerosis with a local increase within the lesion. Adding carotid atherosclerosis to the list of potential battlefields for this cytokine augments the need for further studies of IL-27 in human conditions. The role for IL-27 in inflammasome activation also needs further investigation, and especially studies on the direct effect of IL-27 on NLRP3 assembly are warranted. A combination of *in vitro* experiments, animal models and clinical material would be beneficial in further studies of the immunomodulatory role for IL-27 in atherosclerotic disease.

The present study has some limitations. The number of controls was rather low, particularly when investigating IL-27 expression in carotid plaques. Moreover, we used samples from the common iliac artery of organ donors as non-atherosclerotic vessel controls. At least in our country, these are the best control samples available due to ethical reasons, but ideally we would compare healthy carotid vessels with the atherosclerotic vessels from the same site. Moreover, although we show enhanced capase-1 activity, the lack of western blot data may weaken our findings. Moreover, we were not able to confirm the involvement of STAT1, STAT3 and NFkB activation in the IL-27 induced release of IL-1β in LPS/ATP stimulated THP-1 cells by blocking experiments. On the other hand, the combination of *in vivo* and *in vitro* studies to support a role of IL-27 in atherogenesis, is a strength of the study.

In the present study we show that patients with carotid atherosclerosis are characterized by increased expression of the IL-27 system both in plasma and within the atherosclerotic lesion. Our *in vitro* experiments demonstrate an inflammatory role for IL-27 in monocytes through interaction with LPS and the NLRP3 inflammasome. Our findings may suggest a role for IL-27 in clinical atherosclerosis, potentially mediating inflammatory effects.

## Supporting information

S1 Fig IL-27 does not alter cell death in THP-1 cells stimulated with LPS and ATP. THP-1 cells were primed with LPS (10 ng/mL, 6 hours) and incubated over night with IL-27 (100 ng/mL) before stimulation with ATP (3 mM, 1 hour). Lactate dehydrogenase activity was measured in fresh cell supernatants. Results are representative of three experiments and data are presented as mean and SEM.
(TIF)

S2 Fig Peripheral blood mononuclear cells do not produce IL-27 in response to LPS or NLRP3 inflammasome activation. PBMCs were stimulated with LPS (10 ng/mL) for 6 hours and ATP (3 mM) for 30 minutes. IL-27 release was measured in supernatants with EIA. Data from four independent experiments are presented, as mean and SEM.
(TIF)

S3 Fig IL-27 induces STAT1 and STAT3 phosphorylation in THP-1 cells. THP-1 cells were stimulated with IL-27 (100 ng/mL) for 15 minutes. Totalt protein and phosphorylated (p) STAT1 (**A**), STAT3 (**B**) and IκB (**C**) protein expression were analyzed by western blotting in protein homogenates from cells. Data are presented as mean and SEM of three experiments and representative Western blots.
(TIF)
