# Supplementary figures and images for: Interleukin 27 is increased in carotid atherosclerosis and promotes NLRP3 inflammasome activation
Source: PLoS One. 2017 Nov 27;12(11):e0188387. doi: 10.1371/journal.pone.0188387 (PMC5703457; doi:10.1371/journal.pone.0188387)

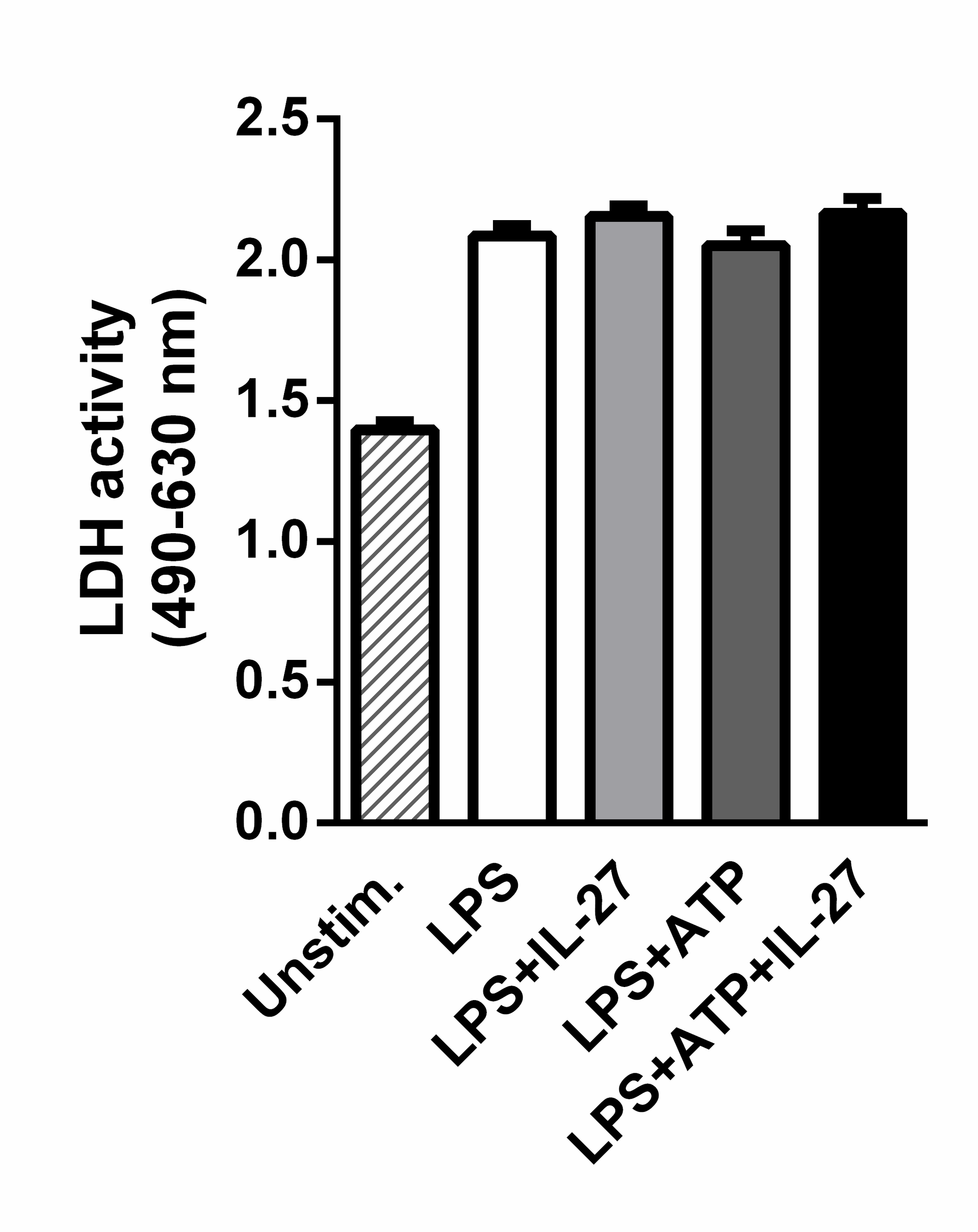

Supplement: S1 Fig — THP-1 cells were primed with LPS (10 ng/mL, 6 hours) and incubated over night with IL-27 (100 ng/mL) before stimulation with ATP (3 mM, 1 hour). Lactate dehydrogenase activity was measured in fresh cell supernatants. Results are representative of three experiments and data are presented as mean and SEM. (TIF) [file pone.0188387.s001.tif]

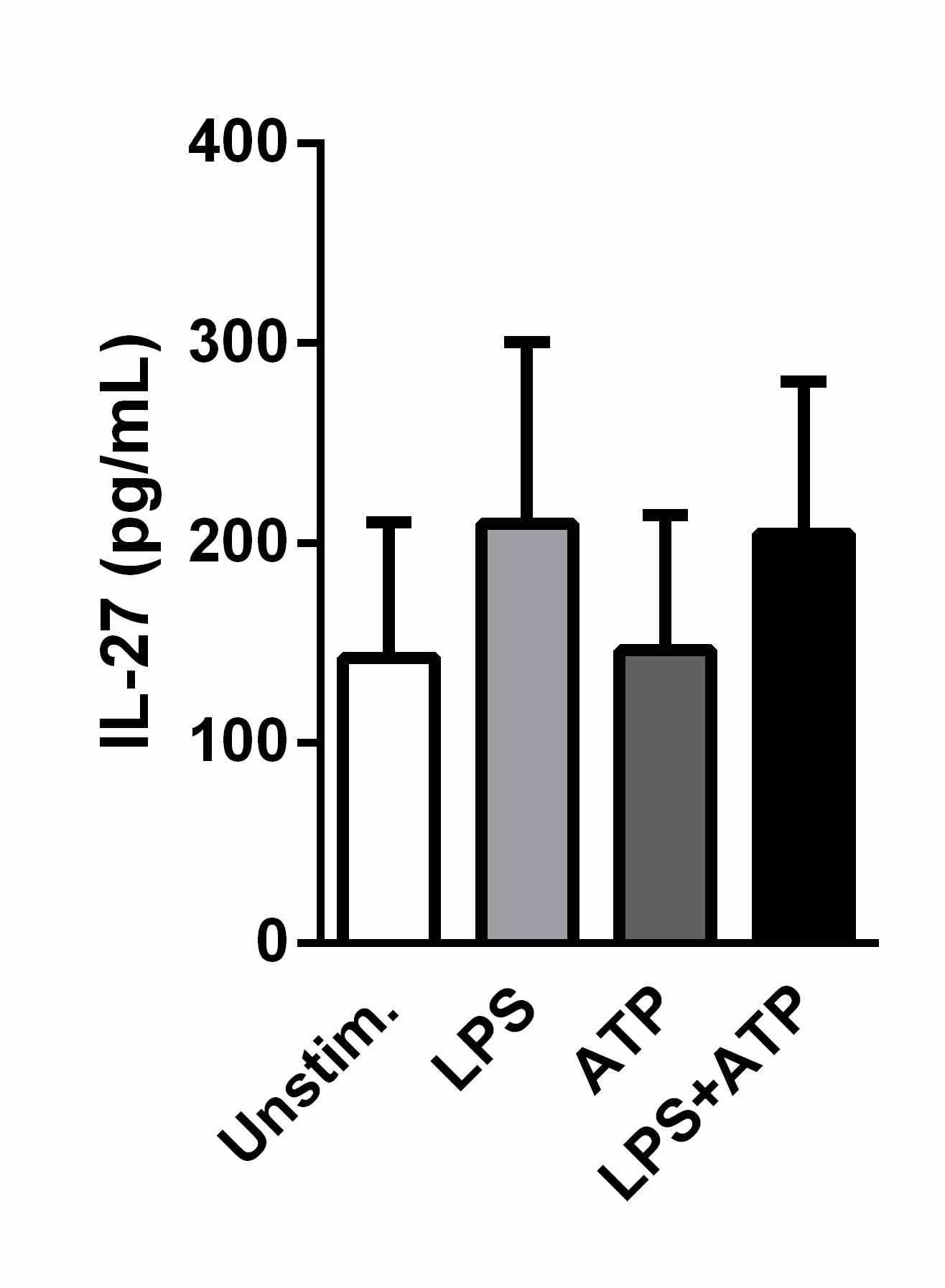

Supplement: S2 Fig — PBMCs were stimulated with LPS (10 ng/mL) for 6 hours and ATP (3 mM) for 30 minutes. IL-27 release was measured in supernatants with EIA. Data from four independent experiments are presented, as mean and SEM. (TIF) [file pone.0188387.s002.tif]

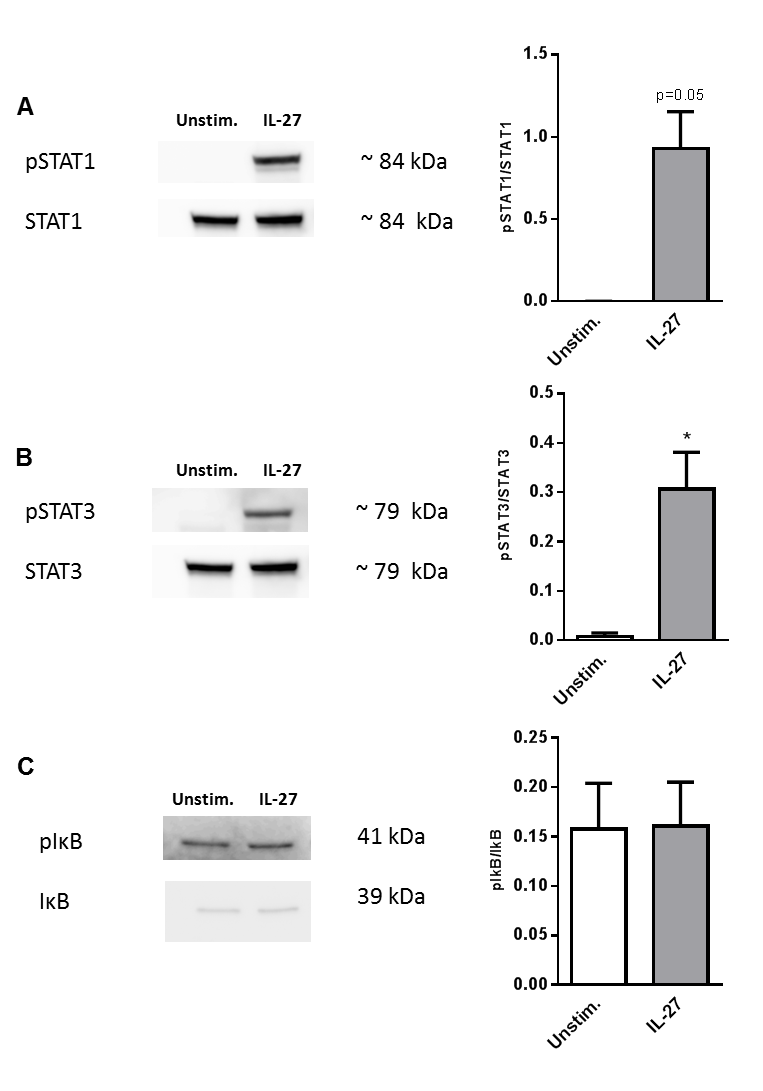

Supplement: S3 Fig — THP-1 cells were stimulated with IL-27 (100 ng/mL) for 15 minutes. Totalt protein and phosphorylated (p) STAT1 (A), STAT3 (B) and IκB (C) protein expression were analyzed by western blotting in protein homogenates from cells. Data are presented as mean and SEM of three experiments and representative Western blots. (TIF) [file pone.0188387.s003.tif]
